# Aceruloplasminemia Presenting With Prominent Psychiatric Symptoms and Neurodegeneration: A Case Report

**DOI:** 10.1002/ccr3.73366

**Published:** 2026-08-21

**Authors:** Ali Almarzooqi, Abdulrahman Almarzooqi, Khalifa Juma, Hamda Kamalboor

**Affiliations:** ^1^ Department of Neuroscience, Dubai Health Rashid Hospital Dubai UAE

**Keywords:** aceruloplasminemia, ceruloplasmin, CP gene, iron overload, neurodegeneration, psychiatric symptoms

## Abstract

Aceruloplasminemia (ACP) often presents with systemic, psychiatric, and neurological features. In this case, diabetes mellitus was diagnosed several years before psychiatric symptoms, which were then followed by neurological decline, which significantly delayed diagnosis. This case highlights the importance of considering metabolic and neurodegenerative causes in atypical psychiatric presentations and expands the genetic spectrum with a novel CP gene mutation.

## 1 Introduction

Aceruloplasminemia (ACP) is a rare autosomal recessive disorder caused by mutations in the *CP* gene, which encodes ceruloplasmin, a multicopper ferroxidase essential for iron homeostasis. Ceruloplasmin oxidizes Fe^2+^ to Fe^3+^, which is required for iron to be loaded onto transferrin and exported from cells. Loss of ceruloplasmin function leads to impaired iron efflux and pathological iron accumulation in the liver, pancreas, retina, and brain. Clinically, ACP is characterized by a triad of diabetes mellitus, retinal involvement, and progressive neurodegeneration [1, 2].

Neuropsychiatric features are common but often underrecognized. Neurological symptoms typically emerge in the fifth decade of life and include cerebellar ataxia, involuntary movements, parkinsonism, as well as psychiatric and behavioral disturbances, and cognitive impairment [2, 3].

Here, we describe an Emirati patient with ACP, in whom diabetes mellitus developed a decade before prominent psychiatric symptoms and subsequent neurological decline. Genetic testing identified a novel homozygous *CP* variant not reported in ClinVar or gnomAD. This case highlights the diagnostic challenges of ACP and underlines the importance of multidisciplinary collaboration in rare neurogenetic conditions.

## 2 Case History/Examination

The patient was a 50‐year‐old Emirati male born to consanguineous parents (first‐degree cousins). There was no family history of similar neurological or psychiatric disorders. He had been diagnosed with type 2 diabetes mellitus 10 years earlier, likely related to pancreatic iron overload, and was treated with sitagliptin and metformin. Glycemic control was poor due to inconsistent adherence.

### 2.1 Psychiatric and Neurological Course (TIMELINE)

- 2013: Diabetes mellitus diagnosed, consistent with an early systemic feature of ACP.
- 2020–2021: He developed psychiatric symptoms including paranoia, persecutory delusions, irritability, crying spells, and simple visual and auditory hallucinations. He was hospitalized for 1 month and discharged on escitalopram, haloperidol, and procyclidine.
- 2021–2022: He developed neurological decline with rigidity, tremors, gait impairment, dysphagia, incontinence, and worsening memory. The Montreal Cognitive Assessment (MoCA) score was 11/30 at first follow‐up.

## 3 Methods

### 3.1 Investigations

Laboratory investigations revealed microcytic anemia (Hb 9.5 g/dL, MCV 76.8 fL) with low serum iron (37 μg/dL), low transferrin (1.99 g/L), and elevated ferritin (2482 ng/mL). Ceruloplasmin and serum copper were markedly reduced (< 0.03 g/L and 20.2 μg/dL, respectively). The patient also had poorly controlled diabetes (HbA1c 12.5%) and dyslipidemia. Liver function tests were largely unremarkable aside from a mild ALT elevation (58 U/L). A peripheral smear showed mild microcytosis and hypochromia; platelets and WBCs were normal.

CSF analysis demonstrated elevated protein (140 mg/dL) and IgG (15.9 mg/dL), with normal glucose (194 mg/dL, consistent with serum hyperglycemia) and no pleocytosis. CSF infectious and autoimmune panels were negative. Thyroid function (TSH and T4) was normal.

**FIGURE 1:**
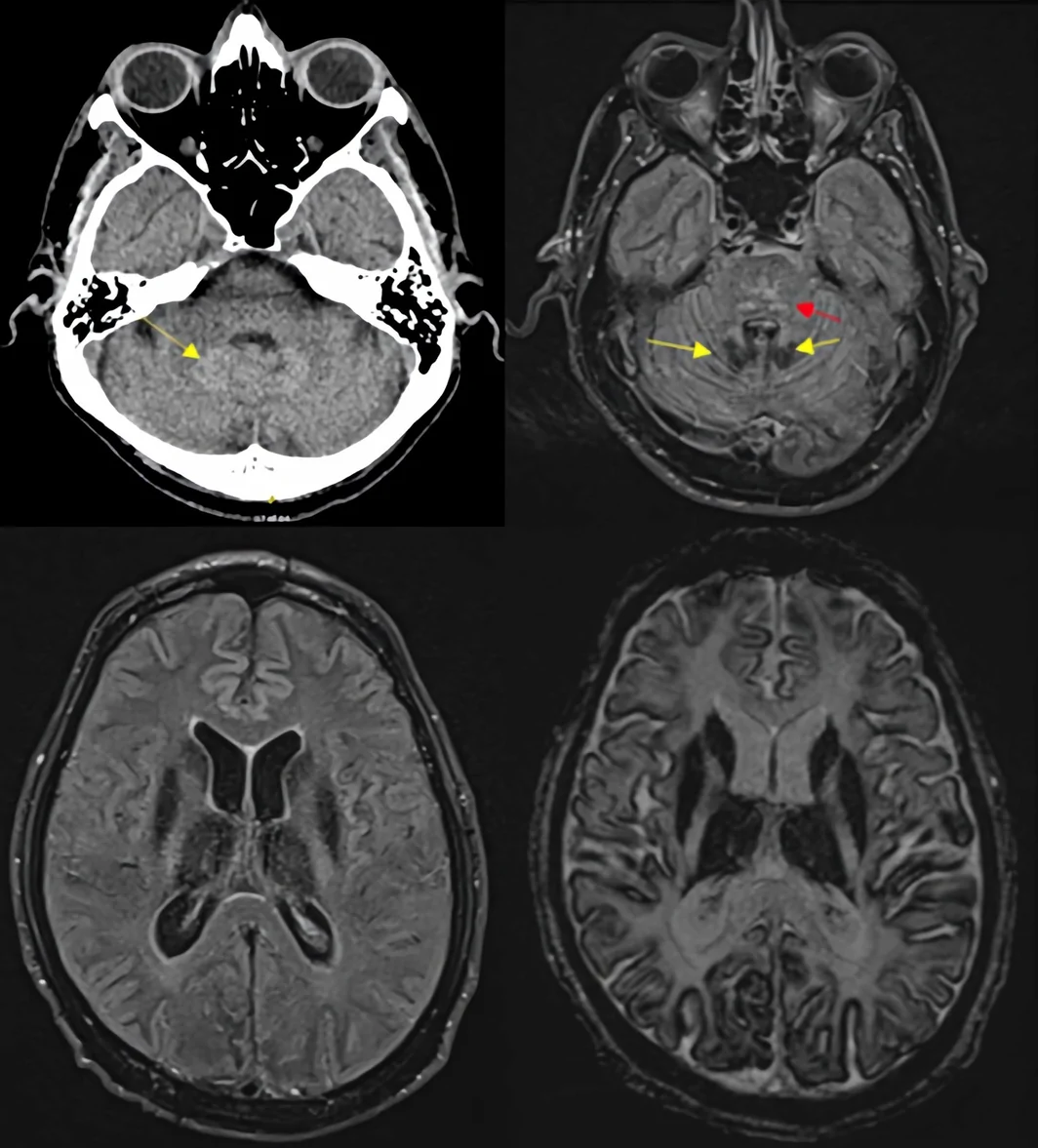
CT and MRI of the brain showed iron deposition in basal ganglia and dentate nuclei. CT brain (axial) showing a hyperdense signal (yellow arrow) in the dentate nuclei (top left). MRI brain FLAIR sequence showing a white matter hyperintensity along the corticospinal pathway extending to the pons (red arrow) and a hypointense signal in the dentate nuclei (yellow arrows) (top right). MRI brain Fluid Attenuated Inversion Recovery (FLAIR) shows hypointense signals in the bilateral thalami, caudate, and lentiform nuclei, as well as a hyperintense signal in the posterior limb of the internal capsule (bottom left). MRI brain SWI confirming iron deposits (bottom right).

**FIGURE 2:**
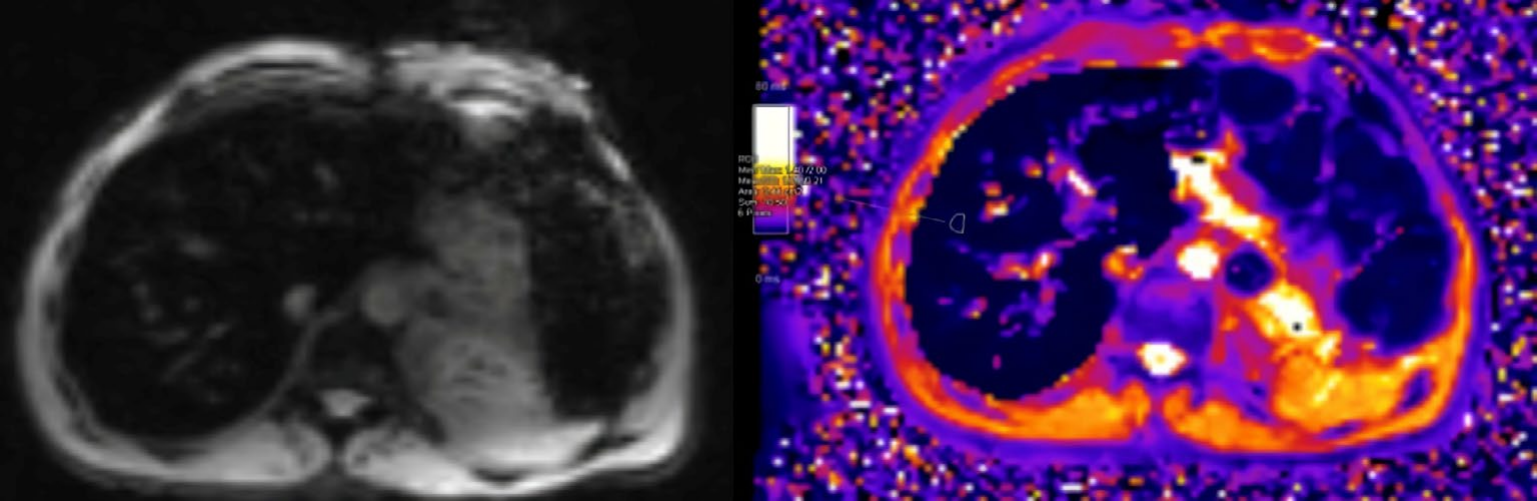
MRI of the abdomen showed iron deposition in the liver. Liver T2* showed liver iron loading was 1.5 ms, corresponding to 8–15 mg/g, which indicates severe liver iron overload [4].

Neuroimaging supported the diagnosis: non‐contrast CT revealed diffuse atrophy with hyperdensities in deep gray matter structures. MRI with T2 and SWI sequences showed hypointensities indicating iron deposition in basal ganglia, thalami, substantia nigra, and cerebellar nuclei, with white matter hyperintensities and brain atrophy (Figure 1). Abdominal MRI demonstrated hepatic and pancreatic iron overload (Figure 2). Although rare cases of ACP with normal MRI findings have been reported in the literature, our patient demonstrated typical iron deposition across brain and systemic imaging, supporting the clinical and genetic diagnosis.

A liver biopsy confirmed hepatocellular siderosis with minimal fibrosis and focal copper accumulation. Hepatocyte nuclei also showed glycogenation. Genetic analysis identified a homozygous pathogenic variant in the CP gene (c.2426‐1G>A), unreported in ClinVar or gnomAD, confirming the diagnosis of aceruloplasminemia.

### 3.2 Differential Diagnosis

The combination of diabetes, microcytic anemia, and neurological symptoms raised suspicion for ACP. However, other inherited metabolic and neurodegenerative disorders were initially considered, including Friedreich's ataxia, mitochondrial syndromes such as MELAS, pantothenate kinase–associated neurodegeneration (PKAN), hereditary hemochromatosis, and multiple sclerosis. Given the patient's movement disorder and psychiatric features, Wilson's disease, Huntington's disease, and autoimmune limbic encephalitis were also part of the differential.

The laboratory profile of microcytic anemia with high ferritin, low serum copper, and absent ceruloplasmin excluded most alternative diagnoses. Neuroferritinopathy was briefly considered but was unlikely given the presence of diabetes, retinopathy, and absent ceruloplasmin. Ultimately, confirmation of a homozygous *CP* gene mutation (c.2426‐1G>A) established the diagnosis of ACP.

### 3.3 Treatment

Management required a multidisciplinary approach. Carbidopa‐levodopa provided partial symptomatic relief of parkinsonism. Iron chelation with deferasirox was initiated but interrupted due to psychiatric and medical complications. Phlebotomy was attempted but subsequently discontinued, and iron chelation was resumed.

## 4 Conclusion and Results (Outcome and Follow‐Up)

After diagnosis, multidisciplinary management was initiated. Symptomatic treatment with carbidopa‐levodopa produced modest improvement in parkinsonian features and temporarily restored functional independence. Iron chelation therapy with deferasirox (20 mg/kg/day) was initiated but was interrupted during episodes of psychiatric relapse and medical complications, during which therapeutic phlebotomy was attempted. Chelation was subsequently resumed and tolerated without further interruption.

By March 2025, serum ferritin had declined to 585 ng/mL. Despite this improvement in systemic iron burden, neurological decline continued. Neurologically, the patient initially stabilized with a Montreal Cognitive Assessment (MoCA) score of 11/30 in 2022, but over the following 2 years he experienced gradual decline with worsening memory, increased irritability, and progression of parkinsonism (shuffling gait, turning en bloc, bradykinesia, and rigidity). Psychiatric relapses continued to complicate adherence and overall disease management (Table 1).

**TABLE 1:** Timeline of clinical progression.

Timeframe	2013 (10 years prior)	2020 (Arrival in Dubai)	Early 2021	Late 2021	Feb 2022
Positive findings	Diagnosed With diabetes mellitus in the Philippines (on sitagliptin and metformin)	Scotoma in right eye Brain fog upon awakening	Paranoid delusions (wife) Delusions of reference (phone surveillance) Hallucinations (visual, auditory)	—	Pain and weakness in right arm Bilateral lower limb weakness Tremors Hypersomnia Irritability/agitation
Negative/associated findings	—	—	Abrupt crying spells Social withdrawal	—	Dysphagia Fecal and urinary incontinence Visual deterioration (R>L) Impaired expressive speech
Comments/interpretation	Early manifestation of ACP due to pancreatic iron overload	Possible early signs of retinal and neurologic involvement	Initial psychiatric presentation—may reflect iron accumulation in basal ganglia/limbic circuits	Likely ongoing noncompliance and progressive disease	Advanced neuropsychiatric syndrome consistent with ACP progression

At the most recent follow‐up, he remained under multidisciplinary care with neurology, psychiatry, endocrinology, hematology, and allied health services. His ongoing medications included subcutaneous insulin for diabetes, carbidopa‐levodopa, procyclidine, deferasirox, and atorvastatin. Despite coordinated management, the patient demonstrated slow but progressive cognitive and motor decline.

## 5 Discussion

We describe a case of a 50‐year‐old Emirati male with ACP who initially presented with diabetes mellitus, followed by psychiatric symptoms and subsequent neurological deterioration. The distinctive aspect of this case was the early presentation of psychiatric symptoms, including paranoia, delusions, and hallucinations, preceding neurological manifestations, which resulted in the delay of identifying the underlying metabolic disorder. The combination of diabetes mellitus and abnormal iron deposition on brain MRI should prompt measurement of serum ceruloplasmin. Low ceruloplasmin levels, characteristic MRI findings, and confirmation of a homozygous CP gene mutation (c.2426‐1G>A) established the diagnosis of ACP. ACP is an extremely rare autosomal recessive disorder of iron metabolism, with an estimated prevalence of approximately 1 in 2,000,000 individuals [1]. Due to its rarity and protean manifestations, ACP is often underdiagnosed or misdiagnosed, resulting in significant diagnostic delays [2]. However, if we increased clinical awareness, especially in cases with low or absent serum ceruloplasmin. This may facilitate earlier recognition and appropriate intervention [3].

Neurologic features typically emerge in mid‐adulthood and may include extrapyramidal signs (tremors, parkinsonism, chorea), cerebellar ataxia, cognitive impairment, and psychiatric disturbances [2, 5]. These usually follow systemic manifestations such as diabetes mellitus, microcytic anemia, and retinal degeneration. However, there is considerable phenotypic heterogeneity in ACP [2, 6]. While most cases have been reported in Japanese or Italian cohorts [6, 7], our patient, an Emirati male, illustrates the broader ethnic distribution of ACP.

Importantly, this case adds to the growing literature that highlights atypical neurologic presentations in ACP. The earliest manifestation in this patient was diabetes mellitus, consistent with the typical disease course [8]. Although less common, psychosis is a recognized feature of ACP, particularly in cases with neurologic onset [2]. Additionally, we highlight the findings of Ketata et al. [9], who observed that male patients with homozygous CP mutations often develop diabetes first, with neuropsychiatric symptoms developing later, mirroring the pattern observed in our patient. Hence, this case broadens the recognized neuropsychiatric spectrum and underscores the importance of considering ACP in middle‐aged individuals with unexplained psychiatric symptoms in the setting of metabolic disturbances.

Genotypically, more than 70 CP gene mutations have been reported [2]. The splice‐site mutation in our case (c.2426‐1G>A) appears to be novel, as it is absent from both ClinVar and gnomAD databases, and to our knowledge has not been previously published. Such mutations expand the known allelic heterogeneity and suggest possible genotype–phenotype correlations. Notably, Ketata et al. [9] also reported a novel CP mutation (c.988A>T) associated with atypical neurologic onset in Tunisian siblings.

In silico prediction tools support the pathogenicity of this variant. SpliceAI, a deep learning model for splicing prediction developed by the Broad Institute, generates Δ scores ranging from 0 to 1, where higher values indicate greater confidence in splice site disruption. Using the SpliceAI lookup tool, our analysis of the patient's variant (c.2426‐1G>A) yielded a Δ score of 0.99 for splice acceptor loss, strongly predicting loss of the canonical splice acceptor site and a high likelihood of exon skipping or intron retention. Pangolin similarly predicted splice site loss with a score of 0.79. Together, these findings suggest that the variant disrupts normal mRNA processing and likely results in a nonfunctional ceruloplasmin protein. SpliceAI results were obtained using the online lookup interface (https://spliceailookup.broadinstitute.org) [10].

Laboratory findings in ACP often include absent ceruloplasmin, low serum copper and iron, and elevated ferritin, consistent with our patient's results [3]. In some cases, ceruloplasmin ferroxidase activity is measured and is typically absent, though this was not assessed in our case. Systemic features, particularly diabetes mellitus (seen in ~68.5% of ACP cases) and microcytic anemia (~80%–86%), are often the earliest clinical clues [6, 8]. Ophthalmologic evaluation revealed central scotomas due to diabetic retinopathy, not retinal degeneration associated with ACP. This distinction is important because retinal pigmentary changes and vision loss have been frequently reported in ACP cohorts, especially in Japanese patients [7], but were absent in this case. Therefore, we focus our discussion on the psychiatric and neurological manifestations that dominated the clinical course.

Our patient's MRI demonstrated typical ACP‐related iron deposition in the basal ganglia, thalami, substantia nigra, and dentate nuclei, consistent with previously reported patterns [11]. This distinguishes ACP from other NBIA disorders, which show more localized or heterogeneous distributions. Although rare cases of ACP with normal MRI findings have been reported [9], the clear radiological iron deposition in our patient supported the clinical and genetic diagnosis.

Treatment of ACP is not standardized and is guided primarily by case reports. Iron chelation remains the mainstay of therapy, with agents such as deferoxamine, deferasirox, and deferiprone commonly used [2]. In our patient, deferasirox was initially administered at 20 mg/kg/day and later reduced without adverse effects. Although deferasirox can reduce hepatic iron, its efficacy in reducing brain iron remains limited [12]. Phlebotomy, attempted in our case, is controversial. Literature suggests that it offers little benefit and may exacerbate anemia or neurologic symptoms due to impaired tissue iron mobilization [2, 9].

Other therapies include fresh‐frozen plasma, which supplies exogenous ceruloplasmin, and zinc or vitamin E to mitigate oxidative stress. Zinc therapy has shown some benefit in symptomatic heterozygous ACP carriers, but evidence remains limited [13].

In summary, this case illustrates an atypical presentation of ACP with prominent neuropsychiatric features and a novel CP mutation. It reinforces key teaching points: (1) MRI typically demonstrates characteristic iron deposition; (2) phlebotomy may not always be beneficial; and (3) resistant psychiatric symptoms in middle age warrant metabolic and genetic evaluation when accompanied by abnormal iron indices.

## Author Contributions

**Ali Almarzooqi:** data curation, investigation, methodology, resources, supervision, validation, visualization, writing – original draft. **Abdulrahman Almarzooqi:** data curation, resources, visualization, writing – original draft, writing – review and editing. **Khalifa Juma:** data curation, resources, visualization, writing – original draft, writing – review and editing. **Hamda Kamalboor:** data curation, investigation, methodology, project administration, resources, supervision, validation, visualization, writing – review and editing.

## Funding

This work was supported by Rashid Hospital, Dubai.

## Consent

The patient provided written informed consent for the publication of this case report and accompanying images, in accordance with the journal's patient consent policy.

## Data Availability

The data that support the findings of this study are available from the corresponding author upon reasonable request.
